# An accelerated framework for high-resolution X-ray holographic reconstruction

**DOI:** 10.1107/S1600577526000275

**Published:** 2026-02-13

**Authors:** Jiarui Hu, Bin Ji, Yu Hu, Lei Wang, Guangcai Chang, Jingyan Shi

**Affiliations:** a Computing Center Institute of High Energy Physics of the Chinese Academy of Sciences 19B Yuquan Road, Shijingshan District Beijing100049 People’s Republic of China; b Multi-disciplinary Research Division Institute of High Energy Physics of the Chinese Academy of Sciences 19B Yuquan Road, Shijingshan District Beijing100049 People’s Republic of China; Paul Scherrer Institute, Switzerland

**Keywords:** X-ray imaging, holography, phase retrieval, high-performance computing, propagation-based phase contrast

## Abstract

X-ray propagation-based phase contrast imaging, a well established imaging technology in synchrotron radiation facilities, enables high-resolution 3D structural reconstruction. Nevertheless, the phase retrieval process required to restore quantitative phase information from holograms remains a significant challenge. Existing software solutions face problems such as performance bottlenecks and limitations in hardware support. Here, we describe a high-performance software named *HiHolo* based on the CUDA-MPI architecture for the holographic regime, and propose three improved iterative phase retrieval algorithms, providing an efficient framework for achieving high-quality holographic reconstruction. Experimental results demonstrate that *HiHolo* achieves 24%–37% performance improvement compared with current mainstream software and exhibits near-linear scalability in multi-GPU systems. The alternating projections with probe algorithm effectively reduces artifacts in traditional empty beam correction by simultaneously optimizing both object and probe wavefields; the extrapolation iteration method enhances the spatial resolution of limited field of view through the computational technique; furthermore, the parallel iterative reprojection optimizes the efficiency of 3D reconstruction, achieving a speedup of about 6–14 times compared with the serial version.

## Abstract 2

*HiHolo*, a high-performance CUDA-MPI software framework for X-ray holographic reconstruction, achieves performance improvement over existing solutions while introducing three enhanced iterative algorithms that effectively reduce artifacts and improve spatial resolution in propagation-based phase contrast imaging.

## 1. Introduction

Propagation-based phase contrast imaging (PBI) has become one of the most widely applied X-ray imaging techniques in synchrotron radiation facilities due to its simple configuration without additional phase-modulating optical elements and excellent phase contrast effects (Snigirev *et al.*, 1995 ▸; Quenot *et al.*, 2022 ▸). Particularly in the holographic imaging regime, PBI can achieve high-resolution, high-contrast 3D structural reconstruction, and provide powerful non-destructive testing tools for multiple fields including materials science, biomedicine and archeology (Diemoz *et al.*, 2012 ▸). However, reconstructing quantitative phase information of samples from holograms, namely the phase retrieval process, remains the core challenge for achieving high-quality imaging (Huhn *et al.*, 2022 ▸). Fourth-generation synchrotron radiation sources are experiencing rapid development worldwide. For instance, the High Energy Photon Source (HEPS), scheduled for completion by the end of 2025 (Jiao *et al.*, 2018 ▸), will provide superior experimental conditions for PBI technology. As one of the three ultra-long beamlines at HEPS, the hard X-ray nano­probe beamline (ID19 beamline) will utilize the extremely low emittance characteristics of the HEPS to focus hard X-rays to nanoscale, forming ultra-high-brightness optical probes (De Andrade *et al.*, 2021 ▸). The HEPS nanoprobe beamline employs multilayer Laue lenses (MLL) as nanofocusing elements with a numerical aperture (NA) of 7.8 mrad; these lenses can focus X-rays to a spot size below 8 nm. As diffraction-limited focusing elements for hard X-rays, MLLs can provide diffraction-limited spot sizes as ‘ideal point sources’. The use of NA beams for hologram recording not only supports high-resolution imaging but also enables the acquisition of larger fields of view (Bajt *et al.*, 2018 ▸). These characteristics make MLLs highly suitable as nanofocusing elements for holographic experimental methods (Zhang *et al.*, 2024 ▸). Therefore, holography will be the primary imaging method for the nanoprobe beamline, while simultaneously posing substantial challenges for data acquisition and processing. In addition to more sophisticated phase retrieval algorithms, there is a pressing need for high-performance software frameworks capable of handling the dramatically increased amount of data and computational complexity.

In recent years, PBI holographic imaging experiments based on multi-distance phase retrieval strategies have been widely applied in synchrotron radiation facilities (Cloetens *et al.*, 1999 ▸; Guo *et al.*, 2018 ▸). However, existing publicly available PBI phase retrieval software is less well developed and exhibits limitations in three areas: computing performance, hardware compatibility and algorithm adaptability. For example, the MATLAB-based *HoloTomoToolbox* (Lohse *et al.*, 2020 ▸), while providing relatively comprehensive phase retrieval algorithms and examples, relies on commercial platforms and lacks multi-GPU parallel support. *PyPhase* (Langer *et al.*, 2021 ▸), developed in Python, features good modular design and can be deployed on computing clusters such as SLURM. However, due to its CPU-based computation, it suffers from insufficient performance when processing large-scale data and has limited algorithm coverage in the holographic regime. These limitations prevent researchers from fully exploiting the superior experimental conditions of new synchrotron sources. As data acquisition rates and resolutions continue to increase, there is an urgent need for user-friendly software solutions designed to leverage modern high-performance computing architectures (Xiang *et al.*, 2024 ▸).

To address the aforementioned issues, this paper introduces a C++-based PBI holographic phase retrieval software called *HiHolo*, which implements a complete workflow from distance calibration, data preprocessing, phase retrieval to tomographic reconstruction. The main contributions of this research include: (i) a high-performance phase retrieval algorithm library implemented with CUDA, supporting multi-GPU parallel processing and significantly enhancing large-scale data processing capabilities; (ii) two improved iterative phase retrieval algorithms: AP with Probe and EPI, which to some extent addresses wavefront distortion and low spatial resolution issues; (iii) a parallel optimized version of the iterative reprojection (IRP) algorithm, substantially improving 3D reconstruction efficiency; (iv) design of user-friendly command-line and Python interfaces, lowering the user adoption threshold and achieving seamless integration with the HEPS computing platform and general users.

First, the theoretical foundations of PBI phase retrieval will be briefly outlined. Next, the software architecture and implementation details will be elaborated, with a particular focus on the enhanced phase retrieval algorithms. Finally, the performance and reconstruction quality of *HiHolo* will be validated through comparative experiments, highlighting its advantages.

## 2. Theory

### 2.1. Propagation-based phase contrast imaging

When an X-ray beam penetrates the examined object, the wavefield experiences a phase shift as well as absorption related to the object’s complex index of refraction. Although these phase changes cannot be directly measured, they are converted into intensity variations during propagation, which can be recorded by detectors (Nugent, 2010 ▸). The modulation of X-rays by the object can be represented by a complex object function,
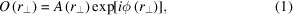
where 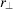 represents the transverse coordinates in the object plane, 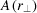 denotes the amplitude attenuation and 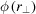 represents the phase shift. After a free-space propagation, the intensity recorded by the detector at propagation distance *z* is
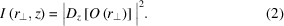
For the case of uniform plane wave illumination, equation (2) could be normalized to unity. *D*_*z*_ represents the Fresnel propagation operator, which can be efficiently computed in Fourier space through
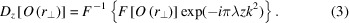
In equation (3), *F* and *F*^−1^ represent the Fourier transform and inverse Fourier transform, λ is the X-ray wavelength and *k* is the wavevector. Based on the propagation distance *z*, wavelength λ and the characteristic length scale *A* of the sample structure, the Fresnel number can be defined as *F*^*A*^ = *A*^2^/λ*z*. According to the Fresnel numbers *F*^min^ and *F*^max^ corresponding to the minimum and maximum image structures, PBI can be categorized into two regimes: direct contrast and holographic (Wu *et al.*, 2008 ▸). This study primarily focuses on the holographic regime, in which measured images exhibit distinct interference fringes and contain rich information across both high and low spatial frequencies.

### 2.2. Iterative algorithm for phase retrieval

For holography, the intensity is the observable but phase information is therefore lost; the key aspect for successful imaging is to recover the lost phase information, a process also termed phase retrieval or phase reconstruction. This is essentially an ill-posed inverse problem that typically requires additional constraints or multiple measurements for effective solution (Fienup, 1982 ▸). Existing phase retrieval methods can be mainly categorized into two types: analytical and iterative. Analytical algorithms such as the contrast transfer function (CTF) are based on physical approximations, offering fast computation but limited accuracy (Paganin *et al.*, 2002 ▸). Iterative algorithms progressively optimize solution quality through repeated projections between the object plane and detector plane, providing broader applicability but higher computational cost (Marchesini, 2007 ▸; Shechtman *et al.*, 2015 ▸). For high-resolution holographic imaging, iterative algorithms typically provide better reconstruction quality especially when prior knowledge about the object information is lacking.

The generic idea of iterative projection algorithms is to alternately apply constraints between the object plane and detector plane. Taking the alternating projections (AP) algorithm as an example, its iterative steps can be expressed as
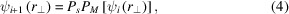
where 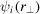 is the exit wavefield at the *i*th iteration, *P*_*S*_ and *P*_*M*_ represent the projection operators for the object plane and detector plane, respectively. *P*_*M*_ is typically a modulus constraint,
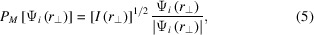
where 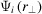 is the value of 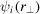 propagated to the detector plane. *P*_*S*_ represents different constraints added according to object characteristics such as pure phase constraints, support constraints *etc*. The convergence of iterative algorithms is closely related to factors including initial guess, constraint conditions and relaxation parameters, and the algorithm may become trapped in local optimal solutions (Bauschke *et al.*, 2002 ▸). Improved algorithms such as hybrid input output (HIO) and relaxed averaged alternating reflections (RAAR) introduce relaxation parameters to avoid falling into local minima while maintaining constraints (Fienup, 1978 ▸; Luke, 2005 ▸). For multi-distance phase retrieval, the AP algorithm can be extended to sequentially apply projections for each distance,
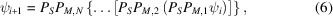
where *P*_*M*, *j*_ represents the modulus projection at the *j*th distance and *N* is the number of distances. Multi-distance strategies can effectively reduce twin-image artifacts in reconstruction and improve reconstruction quality (Guo *et al.*, 2019 ▸).

### 2.3. 3D iterative algorithm

Conventional phase retrieval approaches typically employ a sequential strategy wherein 2D phase retrieval is performed independently for each projection angle, subsequently followed by tomographic reconstruction algorithms to obtain 3D structural information. However, this decoupled methodology fails to exploit the correlations among different projections, potentially compromising reconstruction fidelity (Kudo & Saito, 1991 ▸). The IRP algorithm addresses this limitation by integrating phase retrieval with 3D reconstruction through nested iterative loops incorporating both AP and algebraic reconstruction technique (ART) algorithms (Ruhlandt *et al.*, 2014 ▸). This approach enables direct 3D object structure reconstruction from multi-angle holograms. The principal advantage of the IRP algorithm lies in its inherent enforcement of inter-projection consistency constraints, which effectively mitigate artifacts characteristic of conventional sequential approaches and render it particularly well suited for non-homogeneous sample composition (Ruhlandt & Salditt, 2016 ▸). Nevertheless, the nested iterative framework inherent to this algorithm imposes considerable computational demands, necessitating sophisticated parallel computing architectures for practical application.

## 3. Software architecture and implementation

*HiHolo* is designed for X-ray propagation-based phase contrast imaging in the holographic regime, implementing a comprehensive processing pipeline that comprises four principal components: distance calibration, data preprocessing, phase retrieval and computed tomography (CT). Distance calibration establishes geometric parameters, data preprocessing enhances image quality, phase retrieval recovers phase information from hologram data and CT synthesizes phase retrieval results from multiple angles into 3D structural representations. The software employs C++ as the core development language and integrates CUDA for GPU-accelerated computation, which achieves substantial performance improvements compared with existing open-source packages. Furthermore, it leverages the message passing interface (MPI) to enable multi-GPU parallel processing, which accommodates large-scale data processing requirements. The software provides two interface modes: command-line applications and a Python package, and has been successfully integrated into the HEPS data and computing platform. The documentation and usage examples of HiHolo are available at https://github.com/HuG-Cloud/HiHolo.

### 3.1. Phase retrieval module

The phase retrieval module serves as the central component of the software, implementing the reconstruction process from holograms to quantitative phase information of objects. All algorithms are implemented in CUDA C++, which exploits the GPU parallel computing architecture to deliver order-of-magnitude performance enhancements compared with CPU-based implementations. Through a flexible and extensible framework combined with multi-level performance optimization strategies, the phase retrieval module efficiently processes large-scale datasets generated by modern synchrotron radiation facilities. In terms of computational foundations, *HiHolo* leverages the cuFFT library from NVIDIA CUDA to achieve high-performance Fourier transforms, thereby enhancing wavefield propagation calculations. Simultaneously, it employs the CUDA NPP library for image manipulation operations such as padding and cropping.

The module supports both analytical and iterative phase retrieval algorithms. The analytical algorithms primarily implement the CTF method, which performs direct inversion of phase information in the frequency domain based on linear approximation. This approach is suitable for weak phase objects and offers the advantage of rapid computation (Langer *et al.*, 2008 ▸). However, for samples with significant absorption or phase shifts, the CTF algorithm may generate artifacts. Table 1 ▸ enumerates the currently implemented algorithms, and others such as HoloTIE will be added in the future to enrich the selection. Mainstream iterative algorithms, including AP, RAAR and HIO, progressively optimize reconstruction results through iterative projections between the object plane and detector plane (Krenkel, 2015 ▸). The code of iterative algorithms employs a hierarchical architectural design that explicitly separates wavefield operations, projection operators and solvers. All iterative algorithms share a unified projection framework that achieves interface consistency and efficient algorithm switching through function pointer dispatch strategies. Furthermore, users can select appropriate projection and propagation kernel computation methods according to sample characteristics and experimental requirements. Regarding object plane constraints, the software supports multiple constraint types that can be combined to accommodate diverse samples.

*HiHolo* implements multi-level performance optimization strategies by establishing a two-tier parallel computing model. At the first level, angle-level parallelization, MPI serves as the communication backend and distributes holograms from different angles to separate GPUs for parallel processing. Phase recovery at a certain angle must be performed on a single GPU, and there is no data communication involved. Consequently, this approach will not affect the quality of the reconstruction. Beyond computational task distribution, MPI also optimizes I/O efficiency by integrating parallel file read/write functionality, which enables efficient partitioned data loading and parallel result storage, and thereby significantly reduces data transfer overhead. At the second level, intra-card parallelization, the system leverages CUDA stream technology to achieve fine-grained parallelization of computational tasks, specifically addressing the processing characteristics of multi-defocus-distance holograms. By allocating multiple CUDA streams on the GPU, operations such as boundary processing of holograms, generation of the Fresnel kernel and Fresnel propagation at that distance can be executed concurrently. Each stream processes the holographic data at a specific distance, which effectively masks latency and improves GPU utilization. In terms of memory access optimization, the program extensively utilizes a GPU shared memory mechanism to accelerate matrix computations. While conventional approaches require the creation of complete matrices in global memory when performing operations analogous to row-vector and column-vector multiplication in MATLAB, the optimized method preloads row and column data through shared memory. It reduces global memory access frequency. This strategy is universally adopted in computationally intensive tasks including frequency domain filtering and complex matrix operations, and effectively enhances computational throughput.

For multi-angle reconstruction scenarios, the software employs a modular integration design rather than simple iteration of single-angle phase retrieval functions. This architectural design significantly improves the computational efficiency by identifying and eliminating redundant computations in multi-angle data processing, such as the generation of propagation operators and the application of object plane constraints. Because these operations are equivalently applicable to the measurement data of all angles, the application only needs to perform the relevant calculation once and share the results among different angles. With regard to GPU memory management, *HiHolo* implements sophisticated dynamic memory allocation and deallocation mechanisms. For large-scale multi-angle datasets, a batch processing strategy loads data in batches to GPU memory, which enables the system to handle datasets whose total volume far exceeds single-card memory capacity. Upon the completion of each batch processing, the associated temporary storage space is promptly repurposed for subsequent batches, thereby minimizing the memory footprint. This strategy is particularly effective for high-resolution holograms, as the intermediate wavefield data generated during processing can consume a substantial amount of GPU memory.

### 3.2. Auxiliary module

Distance calibration constitutes a critical component of PBI experimental system configuration and directly influences the accuracy of subsequent phase retrieval. The software follows standard procedures by recording holograms of periodic reference samples at multiple distances, analyzing peak positions in the power spectral density (PSD) to determine magnification factors, and subsequently employing linear fitting to calculate source-to-sample and source-to-detector distances (Bartels, 2013 ▸). This robust and reliable methodology is applicable to geometric parameter calibration in most PBI experimental systems.

The data preprocessing module aims to enhance the quality of raw holographic data through several key procedures, including outlier and artifact removal, dark-field and flat-field corrections and alignment of holograms acquired at different defocus distances. To address horizontal or vertical stripe artifacts commonly encountered in synchrotron radiation experiments, the software implements a linear interpolation-based stripe removal algorithm (Lohse *et al.*, 2020 ▸). Alternatively, stripe removal can be accomplished through frequency domain filtering by excluding corresponding frequency ranges from the PSD. Dark-field and flat-field corrections are performed through standardized normalization procedures using dark-field images recorded without X-ray illumination and flat-field images acquired without objects. The essential image registration step in holographic experiments is implemented using the SimpleITK library, which employs cross-correlation-based registration algorithms optimized through gradient descent methods to determine optimal pixel translations. Both distance calibration and data preprocessing modules extensively utilize matrix operations and image processing functionalities provided by the OpenCV library. The modular design considers processing flexibility, allowing users to selectively apply different preprocessing steps according to specific data characteristics and adjust relevant parameters to achieve optimal results.

The integration of *HiHolo *with *HEPSCT* (Hu *et al.*, 2022 ▸) enables comprehensive CT reconstruction capabilities. *HEPSCT*, developed by the HEPS computing group, is a web-based CT data processing application designed to meet the demands of synchrotron radiation users processing massive X-ray CT data. The backend is implemented with CUDA and Python, achieving 100% GPU acceleration for core reconstruction algorithms. *HEPSCT* offers multiple reconstruction algorithms, with the Grid algorithm capable of finishing 3D reconstruction for 1440 1k × 1k projections within 0.5 s (Fu *et al.*, 2024 ▸). After obtaining the complete 2D reconstruction results calculated by *HiHolo*, *HEPSCT* can be called to execute the 3D structure reconstruction. Both applications have similar user interaction patterns and resource utilization methods, and we will introduce them in the next subsection.

### 3.3. User interface and application deployment

**Figure 1:**
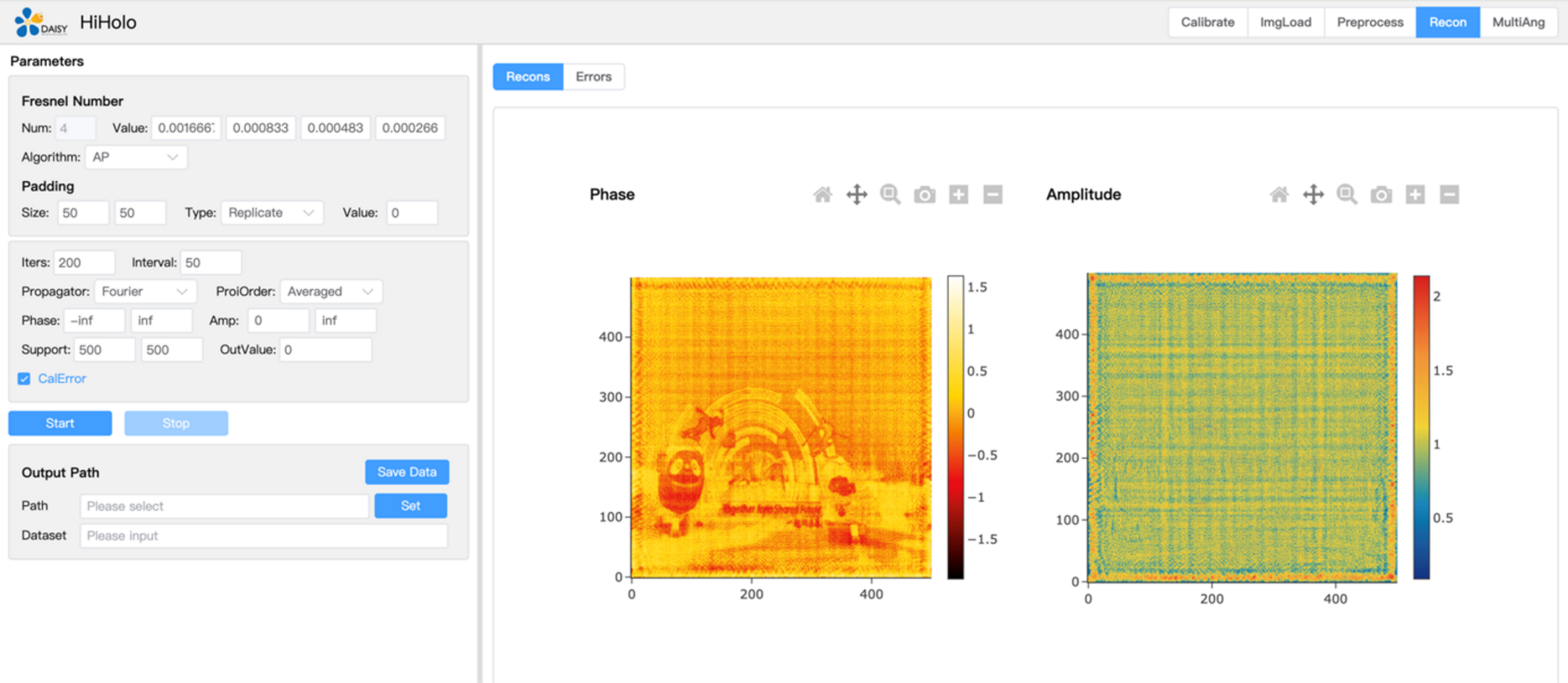
Web interface of *HiHolo* software. The interface displays parameter settings (left) for the AP algorithm including Fresnel numbers and iteration controls, alongside reconstruction results (right) showing phase and amplitude images.

The software provides flexible usage modes through a command-line interface and Python module to accommodate diverse user requirements. Users can control multiple parameters including algorithm selection, constraint conditions and padding types, all of which are configured with reasonable default values that simplify the workflow for novice users while preserving complete customization capabilities for advanced users. Both the command-line tools and Python interfaces adhere to modular design principles, which decompose data processing workflows into independently callable subroutines facilitating integration into larger computational pipelines. Python bindings encapsulate core C++/CUDA algorithms as Python functions and class interfaces, with seamless *NumPy* array conversion support that enables users to directly invoke high-performance computational functions within Python environments. Fig. 1 ▸ shows the user-friendly web-based application under development, which will provide users with more intuitive graphical operation experiences and further reduce barriers to software adoption. Launch of the full version is anticipated when HEPS officially commences operations in early 2026.

Regarding deployment, the software has been successfully integrated into *TORCH* (Hu *et al.*, 2025 ▸), which is developed by the Institute of High Energy Physics Computing Center. This platform offers various computing services, including desktop analysis, interactive analysis and batch analysis, enabling HEPS users to access the computing environment via the web. *TORCH* adopts containerized deployment strategies that dynamically launch container instances based on user resource requirements, with beamline applications such as *HiHolo* pre-packaged in standardized Docker images. Users can select appropriate hardware configurations through the platform interface, and the system automatically allocates corresponding computational resources and launches working environments containing *HiHolo*. This deployment approach eliminates user concerns regarding complex software dependencies and environment configurations, enabling direct access to full functionality through command-line interfaces or Python environments.

## 4. Improved iterative algorithms

### 4.1. AP with probe

**Figure 2:**
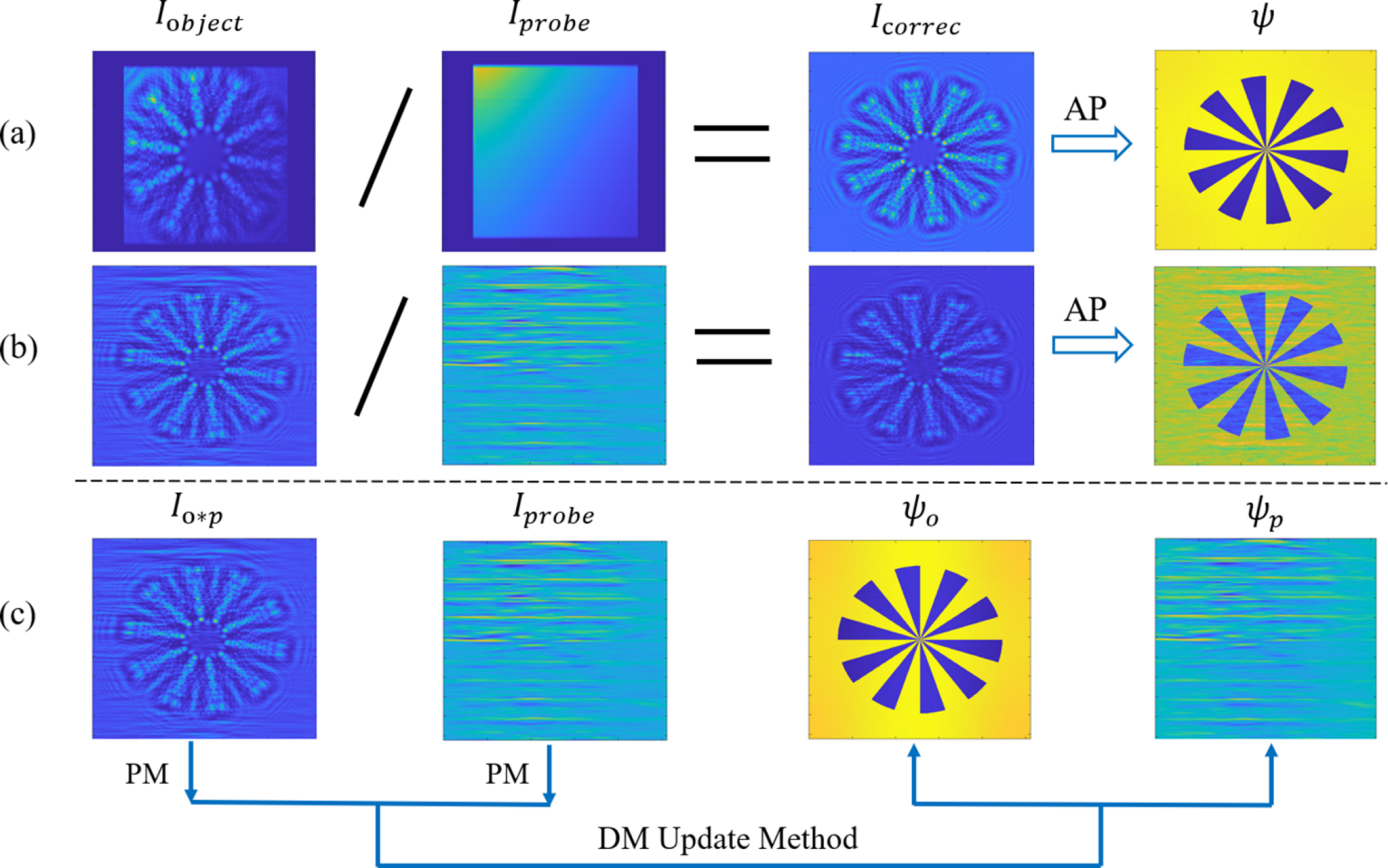
A comparison of empty beam between AP and APWP. (*a*, *b*) Reconstruction effect under an ideal and non-ideal point light source. (*c*) Processing of the object and the probe targets by the APWP algorithm.

In the classical phase retrieval approach, the empty beam correction of the hologram is performed ahead of the phase retrieval as a step in data preprocessing. The influence of probe inhomogeneity is mitigated by dividing the measured intensity with object by the intensity of the empty beam. However, this straightforward correction method performs effectively only under ideal point light source conditions. In practical experiments, due to wavefront errors associated with the probe or significant deviations from an ideal point light source, a conventional preprocessing method can yield substantial errors (Čižmár *et al.*, 2010 ▸; Nikitin *et al.*, 2024 ▸). Figs. 2 ▸(*a*) and 2 ▸(*b*) demonstrate this phenomenon, highlighting the limitations of classical empty beam correction. The AP with Probe (APWP) algorithm proposed in this paper draws inspiration from the difference map (DM) algorithm utilized in ptychography (Maiden & Rodenburg, 2009 ▸). As shown in Fig. 2 ▸(*c*), we integrate flat-field correction into the phase retrieval by treating both the object function and probe as targets to be optimized. This approach facilitates simultaneous phase recovery of object and probe wavefronts. Unlike classic ptychography, this algorithm achieves joint reconstruction of both the probe and object within holographic imaging by incorporating holograms of the probe as additional constraint conditions, without relying on stacking constraints related to scanning positions or excessive data redundancy. In contrast to the traditional AP algorithm, which iteratively updates only the object wavefield, APWP simultaneously optimizes the probe wavefield to better accommodate complex lighting conditions encountered in actual experimental settings. The advantage of the APWP algorithm lies in its ability to simultaneously optimize both the object and probe wavefront during the phase retrieval process. This joint reconstruction approach allows for phase recovery that is more accurate under complex lighting conditions, which would typically result in substantial errors using conventional preprocessing empty beam correction methods.

The mathematical representation of the traditional flat-field correction method is
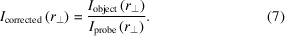
Here *I*_object_ and *I*_probe_ represent the intensity distributions of the object and the empty beam, respectively. In the APWP algorithm, the measured object and the empty beam are treated as measured hologram (P·O) and probe hologram (P), and both hologram (P·O) and probe hologram (P) are used as inputs. The APWP algorithm treats both the object and probe as independent wavefields 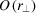 and 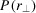, where their combined wavefield is represented by 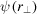 = 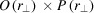. In each iteration, a *P*_*M*_ constraint is applied to the object and probe wavefield. Subsequently, the probe and object function are updated through a separation method aimed at optimizing them simultaneously. During this iterative process, separating the probe and object function from the combined wavefield constitutes a critical step in the algorithm. This procedure primarily relies on principles derived from least-squares optimization. When either one component (the object function or probe) of the overlaid wavefield 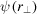 is known, it becomes available to isolate its counterpart using the following formulas:
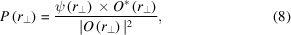

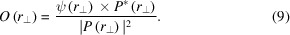

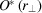
 and 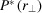 represent the complex conjugations of the object function and the probe, respectively, and the denominator is the strength of the corresponding component. This separation method ensures that the product of the updated object function and the probe closely approximates the wavefield that satisfies the modulus constraint, while maintaining consistency between the two elements.

*Initialization*. Begin with initial estimates for the object function O and the probe P, typically set with uniform amplitude of 1 and phase 0.

*Iteration process*. Repeat the following steps until convergence is achieved or the maximum number of iterations is reached:

– Compute the exit wave as the product of the current object and probe: 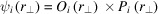. Propagate the exit wave 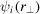 to the detector plane.

– Update its amplitude to match the square root of the measured holograms, retaining the existing phase. Back-propagate this modified wavefield to the object plane to obtain the updated wavefield 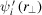.

– Extract an estimate of the probe function from the updated wavefield 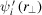 using 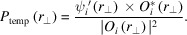


– Propagate the probe 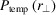 to the detector plane. Apply constraints to 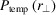 using probe holograms and back-propagate this modified wavefield to the object plane to obtain the updated probe 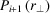.

– From the wavefield 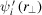, refine the object function by separating it based on the updated probe 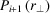, resulting in the updated object *O*_*i*+1_,
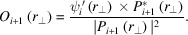
These updated functions *O*_*i*+1_ and *P*_*i*+1_ are then employed to calculate a new exit wave ψ_*i*+1_, completing a single iteration of the APWP algorithm.

To validate the effectiveness of the APWP algorithm, we conducted a reconstruction experiment using a Siemens star pattern as the simulated object. The simulation was performed with a photon energy of 10 keV and a focusing lens with NA of 7.5 mrad and focus length of 1 mm. The sample was placed at a defocus distance of 6 mm. A lens coupled X-ray microscope with square pixels of width 440 nm was placed *L* = 0.23 m downstream of the focus, yielding an effective Fresnel number of 0.0002 according to the Fresnel scaling theorem (Bartels, 2013 ▸); the setup gives an effective pixel size of 13.3 nm. The Siemens star used in the simulation is a pure phase object with a phase ranging from 0 to 1.2 rad, with a diameter of 28 µm. Wavefront distortions characterized by poor flatness were introduced into an idealized incident wavefront to simulate non-ideal illumination. This resulting distorted wavefront was then utilized to rigorously assess the performance of the APWP algorithm under experimentally relevant conditions, the incident wavefront with a phase ranging from 0 to 2.6 rad. We employed 200 iterations of the AP and APWP method for the simulation data.

**Figure 3:**
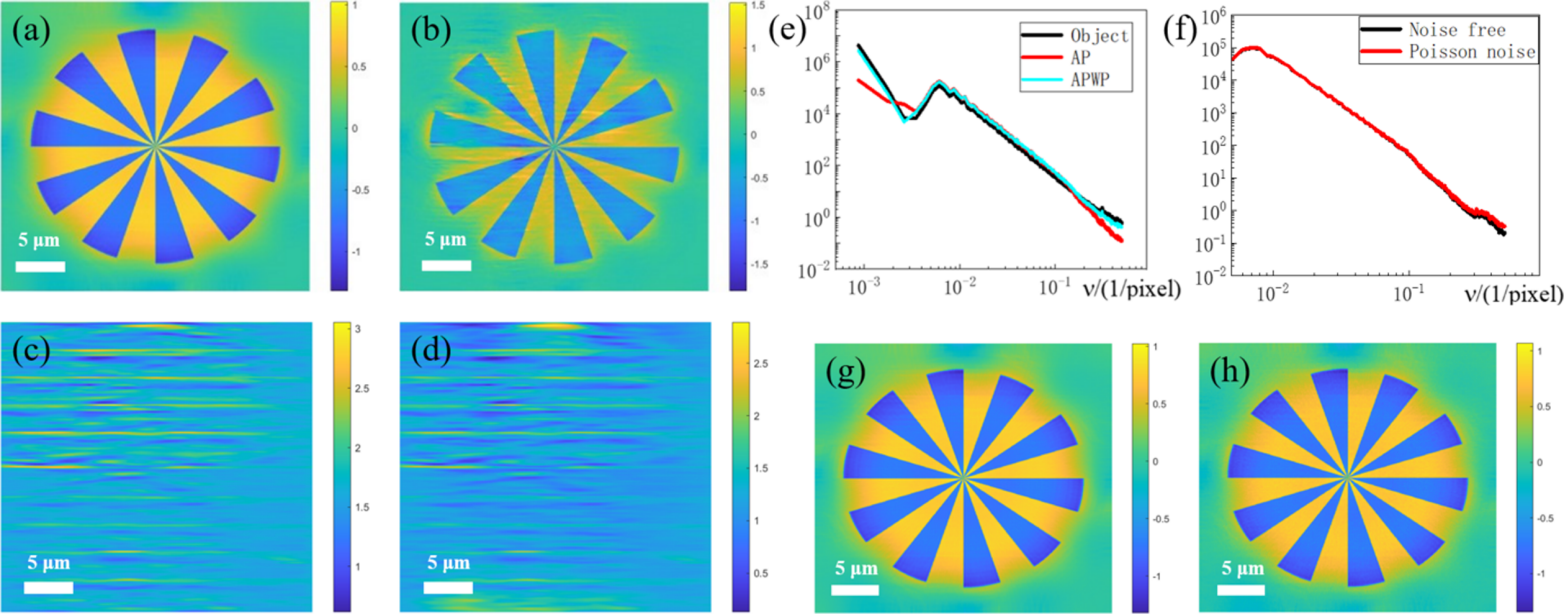
Effect of the APWP algorithm on simulated data. (*a*) The reconstructed phase using APWP. (*b*) The reconstructed phase using flat-field correction and AP. (*c*, *d*) The true phase of the incident wavefront and the result reconstructed by APWP. (*e*) PSDs of the two algorithms and the object. (*f*) PSDs of the latter two conditions. (*g*, *h*) The results obtained using APWP from both noise-free and Poisson-noise holograms.

As demonstrated in Figs. 3 ▸(*a*) and 3 ▸(*b*), the APWP reconstruction exhibits better detail preservation and reduced artifacts compared with the AP reconstruction with traditional empty beam correction, particularly when distortion is relatively obvious. As expected from theory, the reconstructed images are disturbed by the probe (Homann *et al.*, 2015 ▸), and it strongly affects the low spatial frequencies, which can be seen by the PSD results in Fig. 3 ▸(*e*). The figure also shows that the APWP reconstruction and the object are very similar at low spatial frequencies, confirming that the algorithm effectively recovers the structural information of the object. An evaluation of the method’s robustness to Poisson noise was performed. Figs. 3 ▸(*f*) and 3 ▸(*h*) demonstrate the strong robustness of the APWP algorithm against noise. Specifically, the reconstruction resolution is nearly unaffected, with only a minor degradation in high-frequency PSD details observed under noisy conditions.

This result validates the theoretical framework of APWP and its advantages in practical applications: the algorithm is capable of managing non-ideal point light sources and wavefront errors, which is particularly beneficial for synchrotron radiation facilities that utilize intricate optical systems; by considering the actual wavefront of the probe with greater accuracy, it reduces artifacts commonly encountered in traditional approaches, especially at feature edges and in low-frequency details.

### 4.2. AP with extrapolation

In the domain of holographic reconstruction, there exists an inherent contradiction between achieving high resolution and maintaining a large field of view. High resolution necessitates the capture of high-angle scattering signals, which contain critical high-frequency detail information about the object (Thibault *et al.*, 2008 ▸). Conversely, a large field of view requires the detector to encompass a broader spatial range. Due to the physical limitations imposed by detectors, the diffraction beam cannot be completely recorded and high-frequency components are missed, so the two requirements often cannot be fulfilled simultaneously. Traditional approaches including employing larger detectors or stitching together multiple holograms not only escalate experimental equipment costs and radiation exposure but also introduce complications such as stitching errors. To enhance reconstruction resolution within this context, holograms can be extrapolated through computational methods in laser applications (Latychevskaia & Fink, 2013 ▸; Huang & Cao, 2020 ▸). We have applied this approach to the X-ray regime and successfully implemented an improved extrapolation iteration (EPI) algorithm. The EPI algorithm is an effective extension of the AP framework: the primary contribution is including an embedding field around the detector area. The advantage of this algorithm is its ability to enhance the performance of phase retrieval, especially when the sample occupies a high percentage of the imaging field of view, a scenario where conventional methods often struggle.

**Figure 4:**
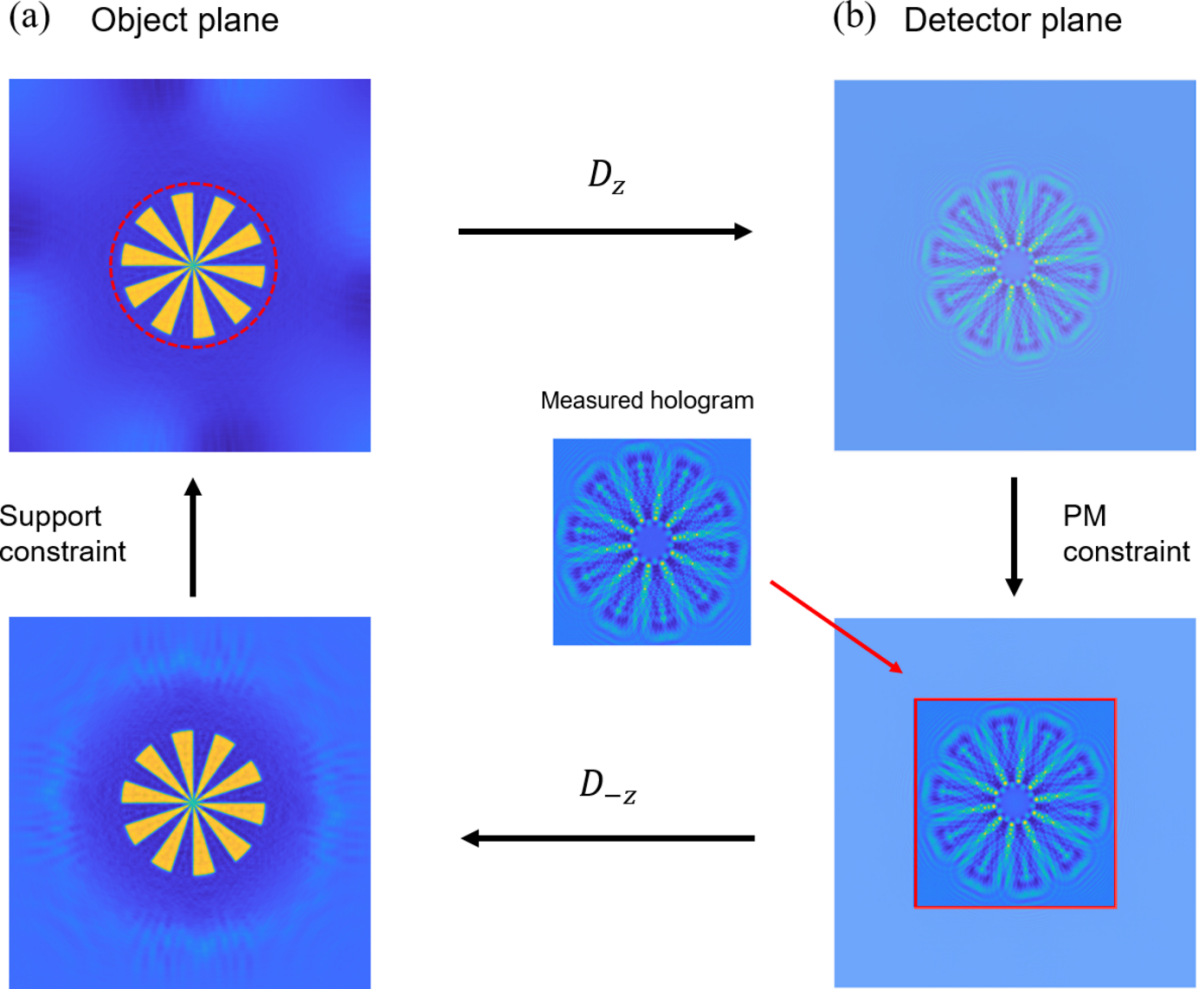
The iterative process of AP with extrapolation algorithm. The method alternates between (*a*) the object plane, where a support constraint is applied, and (*b*) the detector plane, where the PM constraint is applied to the central region of the extrapolated hologram.

The fundamental concept of the EPI algorithm is to leverage the holographic information contained within a limited field of view to infer and reconstruct holographic data in high-angle regions (Rong *et al.*, 2014 ▸), specifically those areas outside the field of view. This approach enhances spatial resolution without an increase in the physical size of the detector. Our innovative research aims to enhance the support constraints, making them more compatible with the underlying physical meaning. In traditional phase retrieval algorithms, whole holograms recorded by the detector are utilized as constraint conditions. In contrast, EPI establishes a computational field of view that exceeds the actual coverage area of the detector, and Fig. 4 ▸ describes the algorithm process. We embed the hologram obtained from this limited field into a larger framework and infer values for outer ring areas while optimizing the overall field of view through an iterative process. A critical step in implementing this algorithm involves performing forward-propagation and back-propagation across the entire extended area during each iteration; however, as shown in Fig. 4 ▸(*b*), the modulus constraint is imposed solely within the region actually recorded by the detector (Latychevskaia & Fink, 2013 ▸). To be specific, we apply the measured holographic data in this region while maintaining unchanged calculation results for extrapolated areas. Following this step, a necessary support constraint and other appropriate restrictions are imposed in the object plane.

Normally the object has a finite size, and a mask is applied to the distribution, ensuring that values beyond a specific area are set to 1. Additionally, a second constraint enforces positive absorption, aligned with the physical principle that the wave’s amplitude should not increase during scattering. As a result, any pixel values with negative absorption are reset to 1. In conventional support constraints, both amplitude and phase outside the support area, *i.e.* the no-object region, are typically assigned a value of 0. This practice does not accurately reflect the actual conditions of ray penetration through areas devoid of objects. Consequently, we have modified the support constraint by assigning a constant phase value of 1 instead of 0 outside the support region, which is comparable with the common strategy of subtracting point-wise 1 and then applying zero-padding, but in a more streamlined, single-step process.

**Figure 5:**
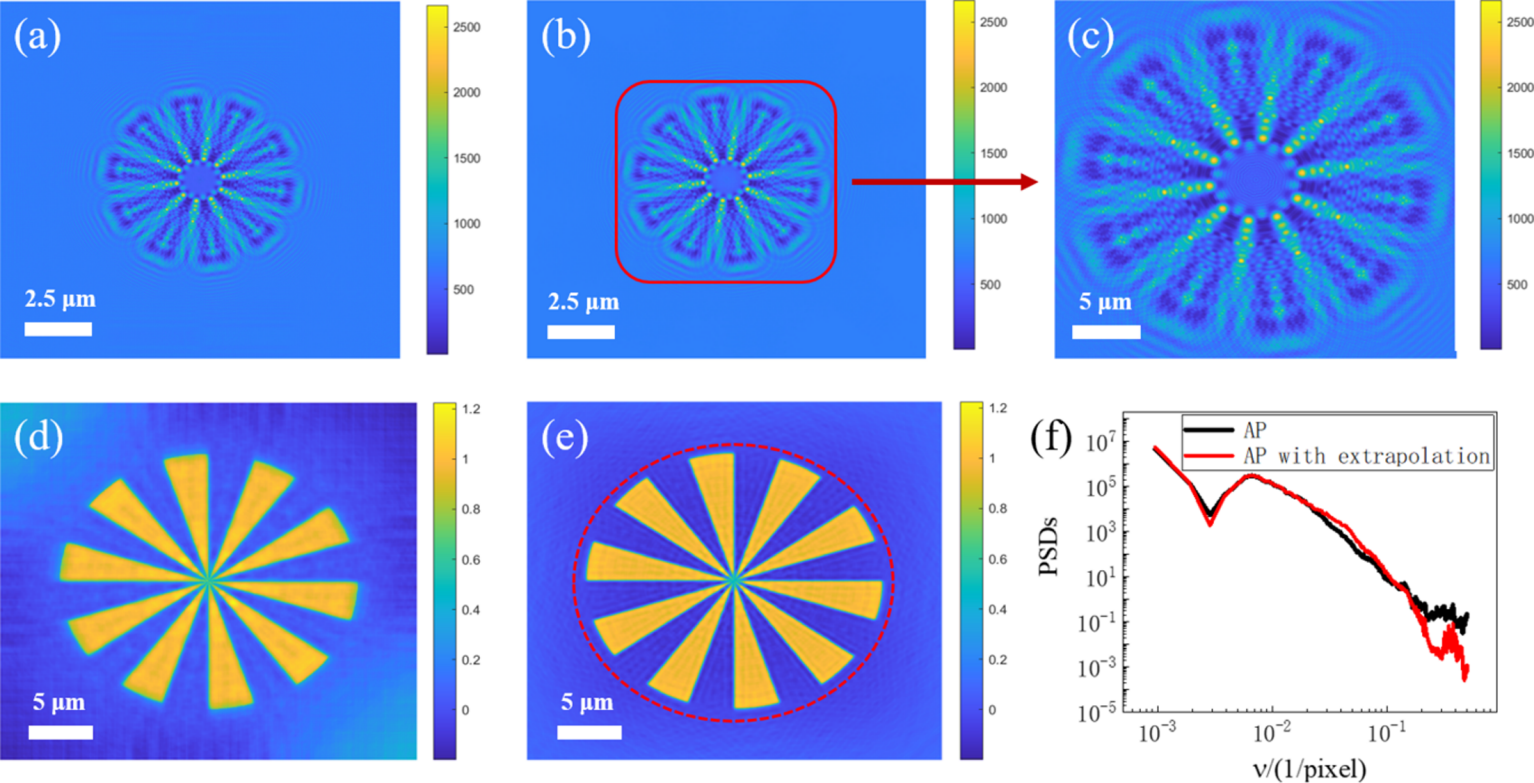
Effect of the EPI algorithm on simulated data. (*a*) Simulated large field of view hologram. (*b*) Hologram computed by the EPI method. (*c*) The central region where the PM constraint is applied. (*d*) The reconstructed phase using AP. (*e*) The reconstructed phase using EPI with the support boundary. (*f*) Azimuthally averaged PSDs of the two results.

To verify the effectiveness of the EPI algorithm, we performed a reconstruction experiment using the same simulated parameters as described in Section 4.1. The results are shown in Fig. 5 ▸. Fig. 5 ▸(*a*) represents a full-field hologram simulated by the Siemens star. Through the EPI algorithm, the limited hologram is computationally extended to a larger field of view. The extrapolated hologram after 260 iterations is shown in Fig. 5 ▸(*b*), and the red outlined region indicates the detector coverage area. Fig. 5 ▸(*c*) displays the central region based on the actual measurements from the detector. As illustrated in Figs. 5 ▸(*d*) and 5 ▸(*e*), the EPI reconstruction exhibits enhanced resolution compared with the AP result, particularly in radial structures where high-frequency information is rich. The quantitative analysis of PSD in Fig. 5 ▸(*f*) proves the performance of the algorithm. At high spatial frequencies, the PSD of the AP with extrapolation decreases to smaller values, indicating a lower noise level of the reconstruction, which is in good agreement with the visual impression of the image.

**Figure 6:**
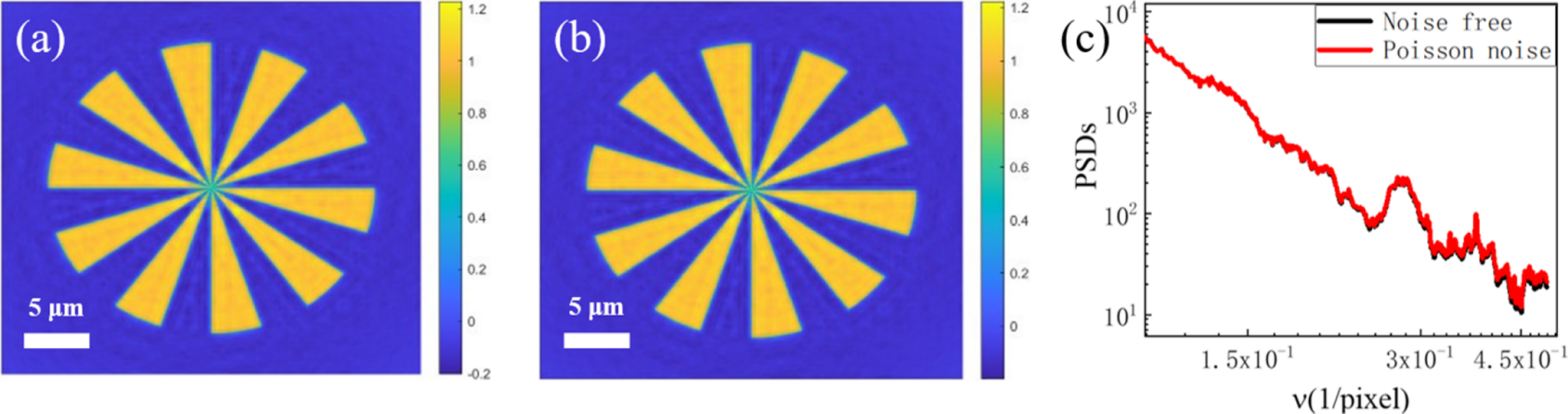
Robustness of the EPI algorithm against noise. (*a*, *b*) The results obtained using EPI from both noise-free and Poisson-noise holograms. (*c*) Azimuthally averaged PSDs of the two results.

As shown in Fig. 6 ▸, the EPI algorithm demonstrated similar robustness against noise, with its reconstruction quality being largely unaffected in terms of resolution and exhibiting only minor attenuation of high-frequency details. The strong robustness common to both the EPI and APWP algorithms originates from the amplification of the object wave by the strong reference beam in the near-field holography experiment (Zhang *et al.*, 2024 ▸). This mechanism enhances the signal-to-noise ratio.

These results not only validate the principle of EPI but also highlight its several notable advantages. Firstly, this algorithm can achieve a significant improvement in spatial resolution without an increase in hardware complexity. Secondly, EPI is well suited for scenarios where the object area is distinctly defined; it performs well with sparse samples or those with clear boundaries. However, the accuracy of extrapolation may be restricted by the quality of the actual measured intensity and the precision of support constraints. Nonetheless, by innovatively integrating the extrapolation technique into the X-ray holographic imaging regime, the EPI algorithm offers a cost-effective and efficient approach for obtaining high-resolution reconstructed phase images.

### 4.3. Parallel IRP

Due to its nested iterative characteristics, the IRP algorithm entails a massive computational burden. The computation time increases nonlinearly with the projection angle and image size, a phenomenon that is particularly pronounced in high-resolution reconstructions. This limitation significantly hinders its practical application (Thompson *et al.*, 2019 ▸). Consequently, this study undertakes parallel optimization of the IRP algorithm and leverages the computational power of modern GPU devices to markedly enhance algorithm performance, which facilitates the application of this high-quality reconstruction method to large-scale datasets within a reasonable time. Based on a heterogeneous parallel architecture that integrates MPI and CUDA, we have developed a parallel IRP (PIRP) algorithm capable of effectively utilizing multiple GPU resources.

**Figure 7:**
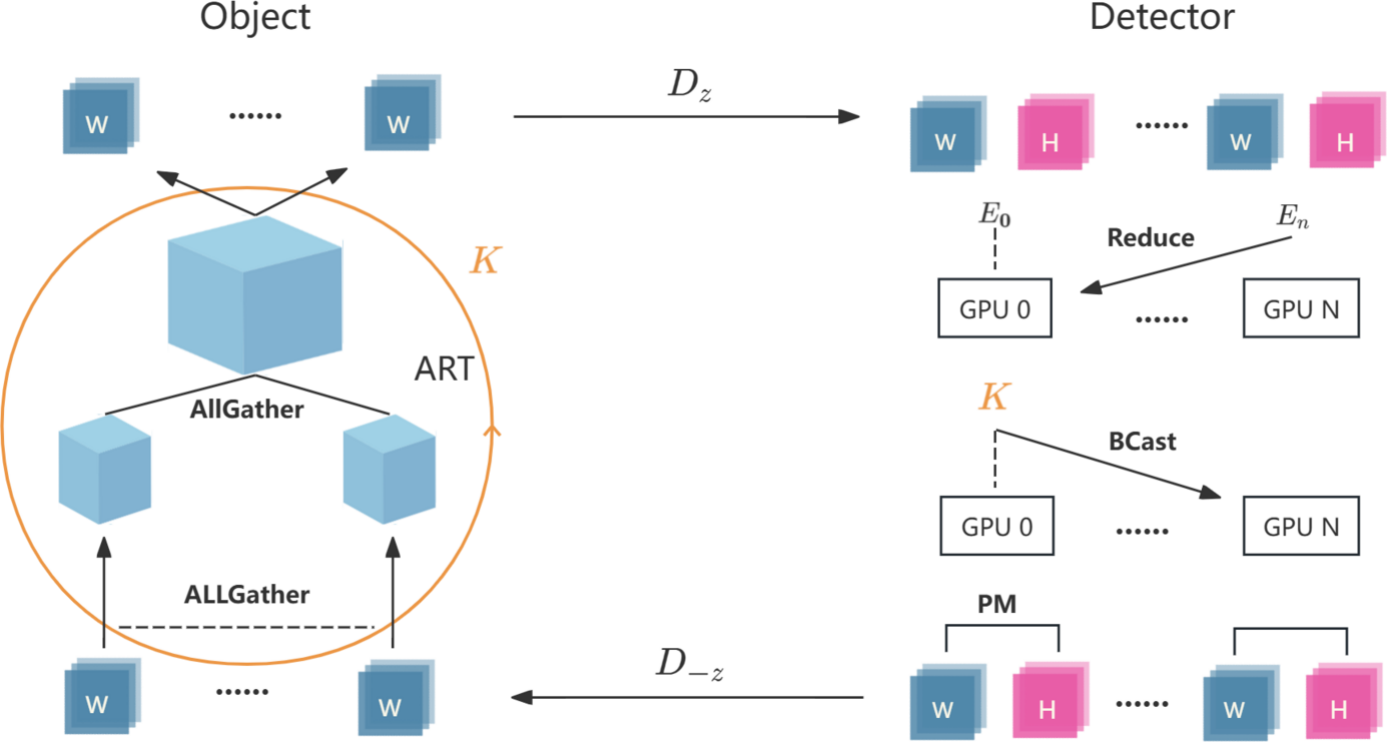
Workflow of the PIRP algorithm. The algorithm distributes 3D reconstruction (ART) and iterative phase retrieval (AP) tasks across multiple GPUs, using MPI communication operations like allgather, reduce and broadcast for efficient data exchange and synchronization.

The fundamental concept of the PIRP is to distribute the computational workload to multiple GPUs, thereby leveraging their capabilities to accelerate computing tasks involved in iterative phase retrieval and CT reconstruction. Fig. 7 ▸ illustrates the primary workflow of the algorithm, which encompasses several key parallel design elements.

*Data distribution and AP algorithm*. A total of *N* angle projection data, *i.e.* the object function wavefields, is evenly distributed across *n* GPUs. Each GPU is responsible for handling the Fresnel forward- and backward-propagation of its assigned data and performs the *P*_*M*_ constraints with the corresponding measured holograms; this segment of the operation does not involve any data communication.

*Voxel decomposition and collaborative reconstruction*. The 3D reconstruction space is evenly decomposed into *n* parts, with each GPU responsible for computing one specific part. During the back-projection phase, each GPU calculates a different section of the 3D grid, and during the forward-projection phase it computes projection data corresponding to its assigned *N*/*n* angles.

*Data communication strategy*. The MPI_Allgather collective communication operation is used for data exchange to execute the ART algorithm, which ensures that each GPU in each round of the inner loop obtains projection data for all angles before back-projection and complete 3D grid data before forward-projection. In addition, error information is communicated using MPI_Reduce and MPI_Bcast in the detector plane to determine whether the termination condition for the inner loop has been met.

*GPU stream concurrency*. In each GPU, we employ CUDA streams rather than the multi-threaded model OpenMP utilized in the original implementation to enable concurrent execution of ART sub-operations. This approach further enhances hardware utilization efficiency.

By partitioning the projection angles and decomposing the 3D reconstruction grid, each GPU only needs to process a subset of the data, which effectively solves the memory limitations of large-scale datasets and makes high-resolution reconstruction possible. Such enhancements greatly boost execution efficiency while preserving the advantages of the original algorithm in reconstruction quality. Although parallel optimization markedly improves performance, the computational and memory requirements may still restrict the applicability of this algorithm for particularly large datasets or ultra-high-resolution reconstructions. Overall, by combining MPI and a CUDA parallel framework, PIRP successfully transforms the computationally intensive IRP into a practical tool for 3D reconstruction, which can be applied to actual large-scale data processing scenarios.

## 5. Experiment evaluation

### 5.1. Performance evaluation

In order to objectively evaluate the performance advantages of *HiHolo* software, we conducted a comparative experiment with the widely used MATLAB-based *Holo­TomoToolbox*. The experimental environment was configured as an Intel Core i7-12700KF CPU with NVIDIA RTX 4070 GPUs. The test employed simulated 4-distance holograms in HDF5 format, which were reconstructed using the classical AP algorithm and default configurations were applied for all other parameters. To ensure a fair comparison, both tools focused exclusively on the phase retrieval step. The execution time for 200 iterations was documented, excluding processes such as parameter parsing and simulation data access.

**Figure 8:**
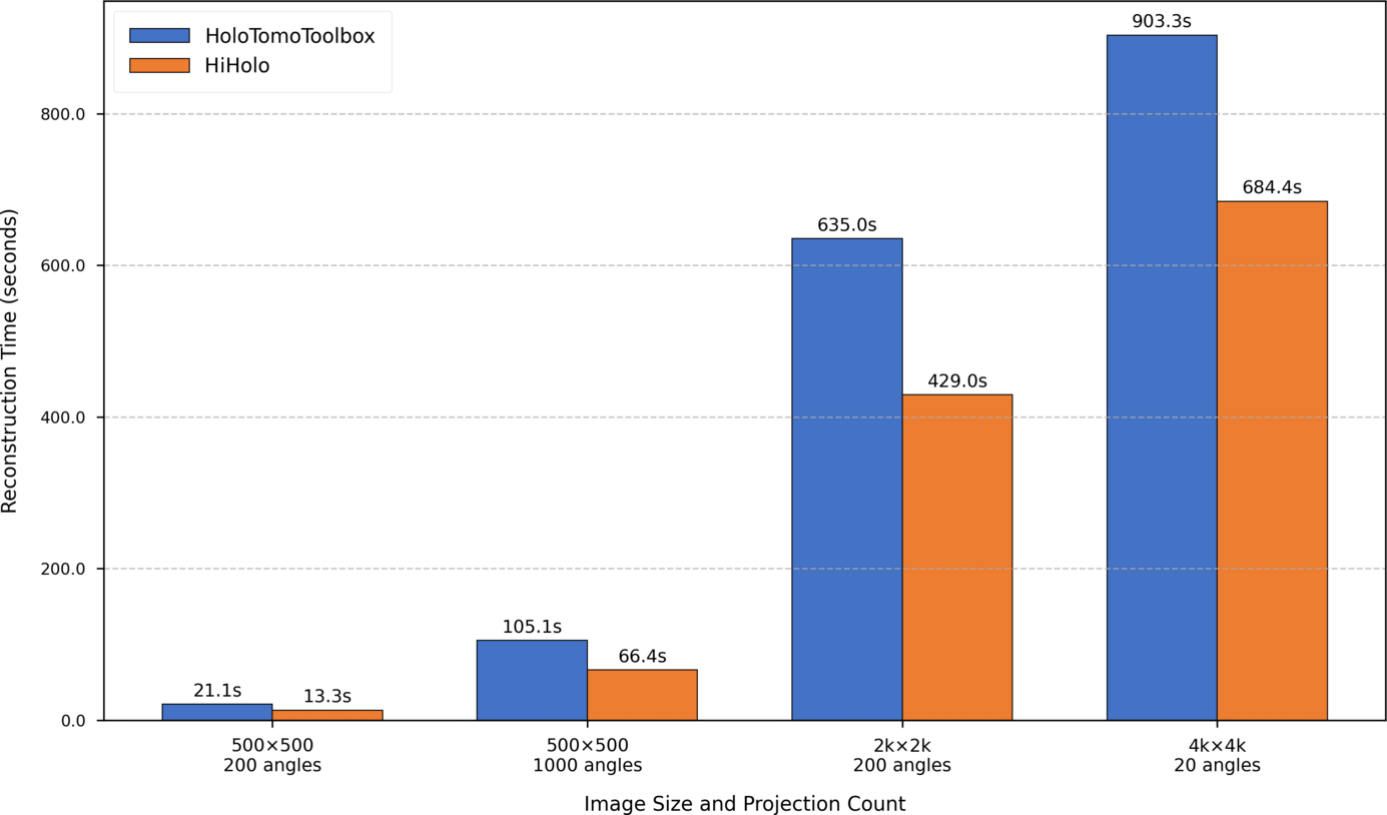
Performance benchmark of *HiHolo* against *HoloTomoToolbox*. The chart compares the reconstruction time on a single GPU for datasets with varying image sizes and projection angles, demonstrating the significant speed advantage of *HiHolo*.

As illustrated in Fig. 8 ▸, *HiHolo* developed on CUDA C++ consistently outperforms *HoloTomoToolbox* in all test scenarios. For images with both a length and width of 500 pixels, *HiHolo* demonstrates an approximate 37% performance improvement compared with *HoloTomoToolbox*. For images with higher resolutions of 2k and 4k, although the performance advantage of *HiHolo* slightly diminishes, it still achieves improvements of 32.4% and 24.2%, respectively. We believe that the observed decline in the proportion of performance improvement when processing 4k resolution images may be attributed to a significant increase in computational load as image size escalates, and the time allocated to the computational part also rises correspondingly. The optimization strategy of *HiHolo* in multi-angle processing mainly focuses on propagators, object plane constraints and GPU memory. Additionally, the vectorized calculation in the MATLAB version shows remarkable performance when handling large matrices. Even so, for complete multi-angle data processing, *HiHolo* exhibits substantial performance enhancements, particularly under common experimental conditions involving 1k or 2k resolution in practical applications. This advancement holds considerable significance for real-time or near-real-time processing of extensive experimental datasets.

**Figure 9:**
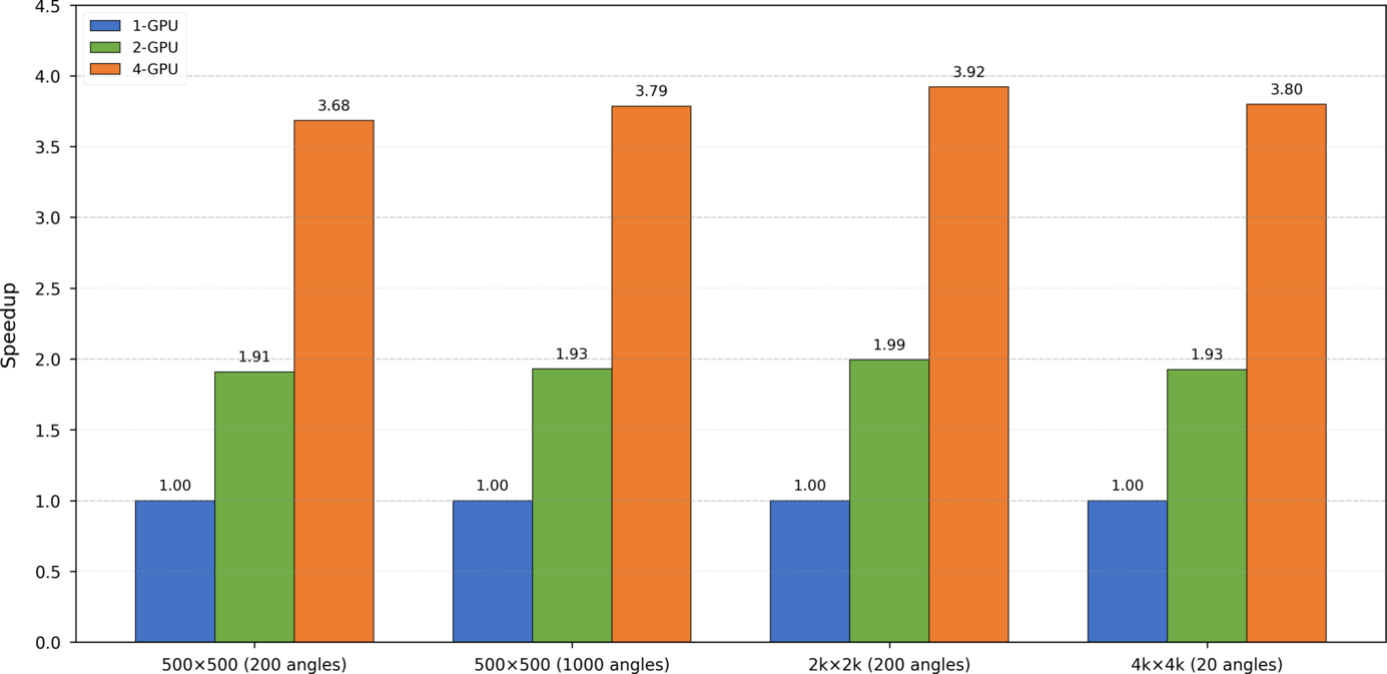
Multi-GPU scaling performance of *HiHolo*. The chart shows speedup with two and four GPUs relative to a single GPU across various datasets. The results confirm near-linear scalability, achieving up to about 3.80× speedup on four GPUs.

To further validate its scaling performance in a multi-GPU environment, *HiHolo* was tested on the above hardware platform. Experimental results in Fig. 9 ▸ demonstrate that *HiHolo* has nearly linear scalability. It is noteworthy that when handling 2k × 2k data, *HiHolo* achieves the best GPU scalability, which is close to the theoretical maximum. Minor nonlinear factors may originate from the overhead of GPU initialization and MPI synchronization communication between data distribution and result collection. The scaling performance of *HiHolo* indicates its high suitability for deployment in large-scale computing clusters and its capability to fully leverage multi-GPU resources for accelerating massive data processing.

**Figure 10:**
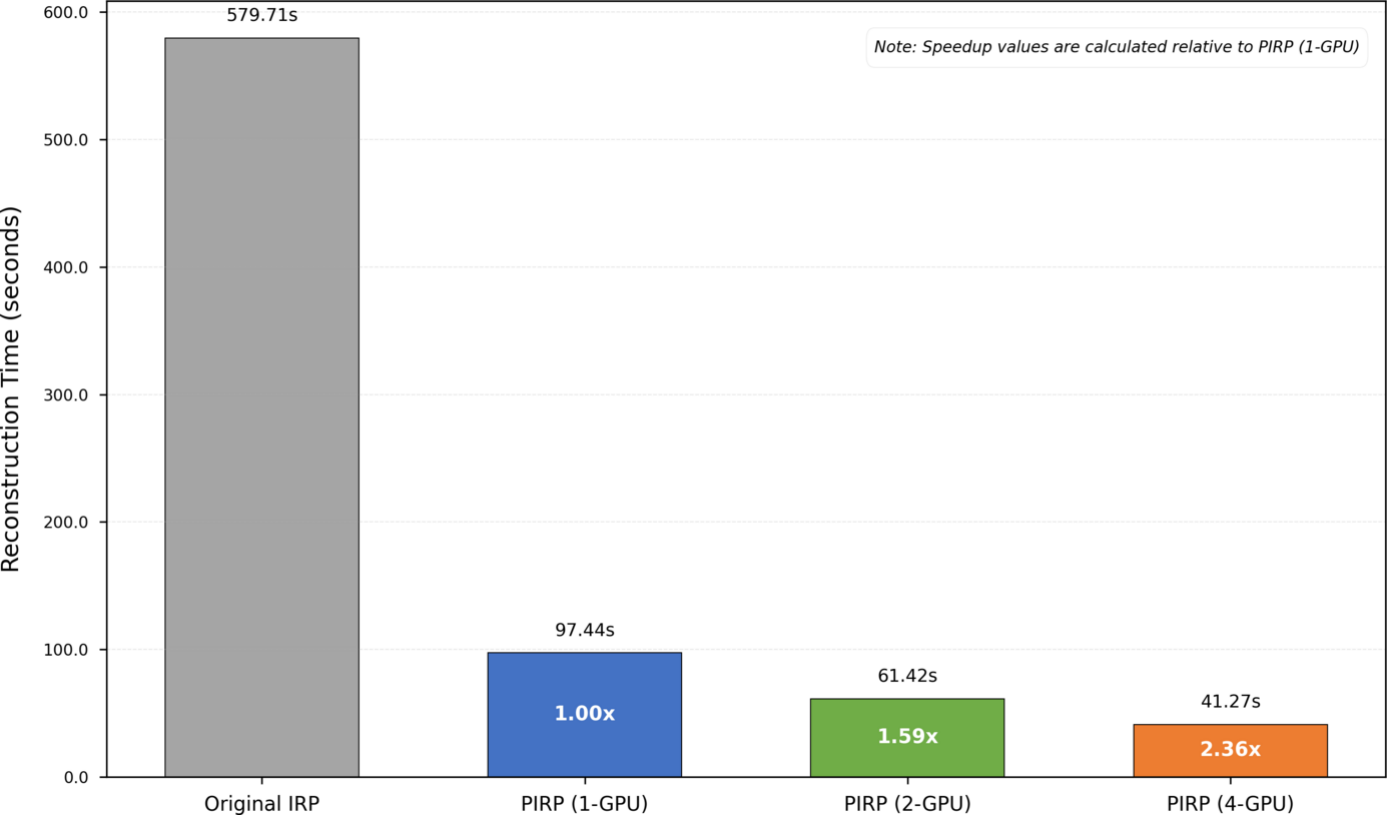
Performance comparison of PIRP and IRP. The chart shows reconstruction times for the original serial IRP versus our PIRP implementation on one, two and four GPUs.

We conducted a comparative test of the PIRP algorithm against the serial version based on the same dataset provided by the original example (Ruhlandt *et al.*, 2014 ▸). It should be noted that the serial version is implemented in C++ with OpenMP, whereas we utilize modules already developed in *HiHolo* to replace the original code, resulting in a greater overall computational load for our implementation. As illustrated in Fig. 10 ▸, PIRP executed on a single GPU is nearly six times faster than the original IRP algorithm. This notable improvement can be primarily attributed to the complete parallelization of constraint computation and CUDA acceleration of ART projection operations. When scaled to a multi-GPU system, PIRP exhibits commendable yet nonlinear scalability. This is mainly due to MPI communication overhead, as GPUs must exchange all the projection data and the whole 3D reconstruction results. These communication costs tend to escalate with an increasing number of GPUs involved, so it is reasonable that some performance loss occurs. Despite this limitation, the speedup from the original algorithm to the execution on four GPUs still reaches approximately 14 times, thereby enhancing the practicability of the IRP algorithm.

### 5.2. Reconstruction quality evaluation

**Figure 11:**
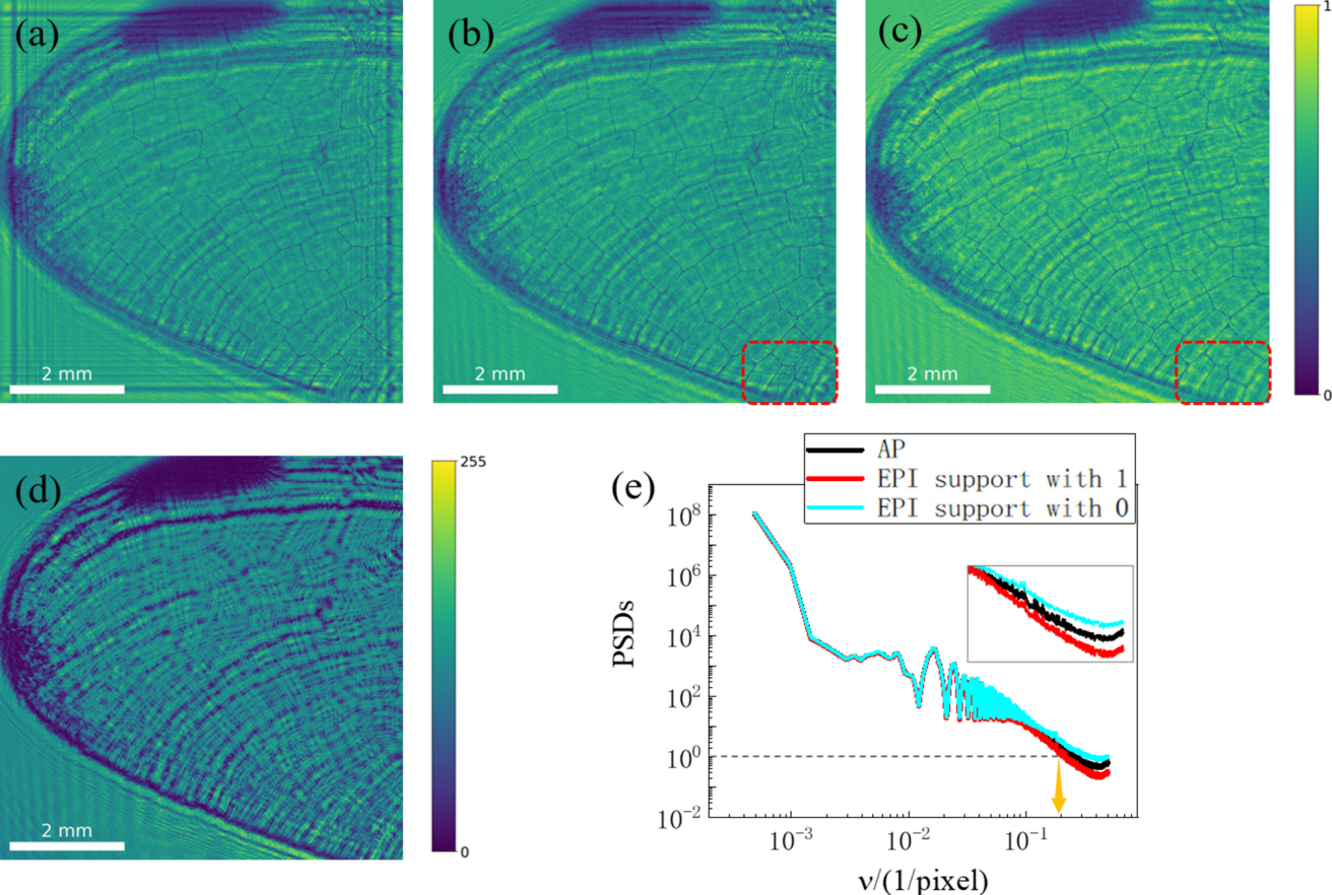
Hologram and reconstructed results based on different phase retrieval methods. (*a*, *b*) The reconstructed results using the EPI method with support 0 and 1. (*c*) The reconstructed result using AP. (*d*) Hologram captured by the detector. (*e*) Azimuthally averaged PSDs of the three results.

A laser at the wavelength of 532 nm was employed to illuminate a dragonfly wing to form the inline hologram on the CMOS detector. The pixel size of the CMOS detector is 3.45 µm and the distance from the object to the CMOS is about 74.9 mm. Therefore, the effective Fresnel number is 2.987 × 10^−4^ according to the Fresnel scaling theorem (Bartels, 2013 ▸). Fig. 11 ▸ shows the hologram and reconstructed results using different phase retrieval methods including the proposed EPI algorithm. Setting the support outer region to 0 will introduce significant artifacts, as seen in Fig. 11 ▸(*a*) which includes horizontal and vertical stripes at the boundaries. A comparison of the details in the red boxes of Figs. 11 ▸(*b*) and 11 ▸(*c*) demonstrates that the EPI method reconstructs finer structures. Fig. 11 ▸(*e*) presents the quantitative analysis of the azimuthally averaged PSDs of different reconstructed results. The result of using a support constraint of 0 is disturbed by noise, which confirms that this constraint choice reduces the reconstruction quality. For the comparison between EPI with 1 support and AP, the EPI reconstruction shows a stronger decrease to smaller values at high spatial frequencies. This indicates a better reconstruction performance, which is consistent with the visual impression of a clearer image. The PSD for the EPI with support 1 shows a cross-over to the noise level at approximately 0.2 cycles per pixel, corresponding to a half-period resolution of 6.9 µm.

## 6. Conclusion

We have developed a high-performance *HiHolo* software and propose three improved iterative phase retrieval algorithms to address the technical challenges in the X-ray PBI holographic regime. *HiHolo* is implemented using a C++/CUDA/MPI architecture, covering the entire processing workflow of holotomography experiments and offering users multiple interface options. Performance evaluations indicate that *HiHolo* achieves a performance improvement ranging from 24.2% to 37% compared with *HoloTomoToolbox*, and has near-linear scalability in a multi-GPU system. Additionally, the PIRP algorithm significantly boosts the efficiency of 3D phase retrieval through a hybrid parallel architecture. Reconstruction quality evaluations show the APWP algorithm effectively reduces the influence of wavefront distortion by integrating empty beam correction into the phase retrieval iteration process. The EPI algorithm employs extrapolation techniques to extend the effective information of holograms, thereby enhancing spatial resolution. Future work will focus on adapting to additional acceleration devices such as the deep computing unit (DCU) of Sugon and developing a client version based on *Qt* to provide a more diverse user experience. With the operation of fourth-generation synchrotron radiation sources like HEPS, *HiHolo* is poised to offer support for high-quality PBI holographic reconstruction while providing users with convenient and efficient data processing tools.

## Figures and Tables

**Table 1:** List of the phase retrieval algorithms implemented in *HiHolo* and their original references

Algorithm	Module	References
Contrast transfer function	reconstruct_ctf	Cloetens *et al.* (1999 ▸)
Relaxed averaged alternating reflections	reconstruct_iter	Luke (2005 ▸)
Hybrid input output	reconstruct_iter	Fienup (1978 ▸)
AP with probe	reconstruct_iter	Nikitin *et al.* (2024 ▸)
Extrapolation iteration	reconstruct_epi	Latychevskaia & Fink (2013 ▸)
Parallel IRP	reconstruct_pirp	Ruhlandt *et al.* (2014 ▸)
