# An accelerated framework for high-resolution X-ray holographic reconstruction. Corrigendum

**DOI:** 10.1107/S160057752600319X

**Published:** 2026-03-26

**Authors:** Jiarui Hu, Bin Ji, Yu Hu, Lei Wang, Guangcai Chang, Jingyan Shi

**Affiliations:** a Computing Center Institute of High Energy Physics of the Chinese Academy of Sciences 19B Yuquan Road, Shijingshan District Beijing100049 People’s Republic of China; b University of Chinese Academy of Sciences 19A Yuquan Road, Shijingshan District Beijing100049 People’s Republic of China; c Multi-disciplinary Research Division Institute of High Energy Physics of the Chinese Academy of Sciences 19B Yuquan Road, Shijingshan District Beijing100049 People’s Republic of China; Paul Scherrer Institute, Switzerland

**Keywords:** X-ray imaging, holography, phase retrieval, high-performance computing, propagation-based phase contrast

## Abstract

Corrigendum to the article by Hu *et al.* [(2026). *J. Synchrotron Rad.***33**, 454–466].

An author affiliation was missing from the address listing in the paper by Hu *et al.* (2026 ▸). The correct address listing is shown above.
